# Development and evaluation of indirect enzyme-linked immunosorbent assay using recombinant dense granule antigen 7 protein for the detection of *Toxoplasma gondii* infection in cats in Thailand

**DOI:** 10.14202/vetworld.2022.602-610

**Published:** 2022-03-12

**Authors:** Eukote Suwan, Piangjai Chalermwong, Rucksak Rucksaken, Metita Sussadee, Sarawan Kaewmongkol, Ruenruetai Udonsom, Sathaporn Jittapalapong, Bandid Mangkit

**Affiliations:** 1 Department of Veterinary Technology, Faculty of Veterinary Technology, Kasetsart University, Bangkok, Thailand; 2 Department of Protozoology, Faculty of Tropical Medicine, Mahidol University, Bangkok, Thailand; 3 The Faculty of Veterinary Technology, Kasetsart University, Bangkok, Thailand

**Keywords:** cats, *GRA7*, indirect enzyme-linked immunosorbent assay, recombinant protein, serodiagnosis, *Toxoplasma gondii*

## Abstract

**Background and Aim:**

*Toxoplasma gondii* is recognized as a zoonosis causing toxoplasmosis in animals globally. Cat is a definitive host of *T. gondii* and sheds oocyst through feces, which can infect human beings and animals through contaminated food ingestion. A precise diagnostic test is essential to prevent *T. gondii* infection in both humans and animals. This study aimed to develop and evaluate the pETite-dense granule antigen 7(GRA7)-based indirect enzyme-linked immunosorbent assay (ELISA) to detect *T. gondii* infection in cats.

**Materials and Methods:**

*T. gondii*-*GRA7* was cloned and expressed in the Expresso^®^small ubiquitin-related modifier (SUMO) T7 Cloning and Expression System. The recombinant pETite-GRA7 was purified using HisTrap affinity chromatography and confirmed using Western blot analysis. The recombinant protein was used to develop and evaluate the indirect ELISA for *T. gondii* infection detection. In total, 200 cat sera were tested using pETite-GRA7-based indirect ELISA and indirect fluorescent antibody test (IFAT). The statistical analysis based on Kappa value, sensitivity, specificity, positive predictive value, negative predictive value, *χ*^2^ test, and receiver operating characteristic (ROC) curve was used to evaluate the performance of the test.

**Results:**

A 606 bp *GRA7* polymerase chain reaction (PCR) product was obtained from *T. gondii* RH strain genomic DNA. The gene was cloned into the pETite™ vector and transformed to HI-Control *Escherichia coli* BL21 (DE3) for protein expression. Approximately 35 kDa of recombinant pETite-GRA7 was observed and Western blot analysis showed positive bands against anti-6-His antibody and positive-*T. gondii* cat serum. A sample of 0.5 μg/mL of pETite-GRA7 was subjected to indirect ELISA to detect *T. gondii* infection in the cat sera. The results showed sensitivity and specificity of pETite-GRA7-based indirect ELISA at 72% and 96%, respectively. An acceptable diagnostic performance was characterized by high concordant results (94%) and substantial agreement (Kappa value=0.65) with IFAT. The seroprevalence levels of ELISA and IFAT were 10% and 9%, respectively, and were not significantly (p>0.05) different. The expected performance of ELISA at different cutoff points using the ROC curve analysis revealed 89% sensitivity and 92% specificity at the cutoff value of 0.146, with a high overall assay accuracy (area under the curve=0.94).

**Conclusion:**

In this study, the pETite™ vector, N-terminal 6xHis SUMO fusion tag, was used to improve the solubility and expression level of GRA7. The recombinant pETite-GRA7 showed enhanced protein solubility and purification without special condition requirements. This pETite-GRA7-based indirect ELISA showed high concordant results and substantial agreement with IFAT. ELISA revealed an acceptable sensitivity and specificity. These initial data obtained from cats’ sera demonstrated that pETite-GRA7-based indirect ELISA could be a useful method for local serological diagnosis of *T. gondii* infection in cats in Thailand.

## Introduction

*Toxoplasma gondii* is an obligate, intracellular protozoan of almost all warm-blooded animals [1]. It is recognized through zoonosis and causes toxoplasmosis involving fetal malformations, premature birth, and nervous system disorder, with one-third of the world’s population being at risk of catching this disease [1-5]. Humans become infected either through ingestion of *T. gondii* tissue cysts in improperly half-cooked meat, some stages-tachyzoites in milk [6,7], or oocyst from feline fecal contamination [8,9]. Cats are the final host of *T. gondii* [2], and their feces are an important source of infective stage-sporulated oocysts that can infect various animals, including birds and humans [10,11]. The oocysts are difficult to detect using feline fecal examination since they are shed from the presence of definitive hosts in a very short time [12,13]. Thus, serological tests such as enzyme-linked immunosorbent assay (ELISA), the latex agglutination test (LAT), the dye test (DT), the modified direct agglutination test (MAT), and indirect fluorescent antibody test (IFAT) are useful [14] to detect anti-*T. gondii* antibodies, particularly when the specific clinical sign is absent. Several serodiagnostic tests based on native antigen prepared from tachyzoites are difficult to standardize, possibly caused due to problems during the production and purification processes, leading to inadequate specificity [15]. Thus, to improve the serological tests, the native antigen is replaced with recombinant antigen to overcome the limitations of the former.

Several antigens of *T. gondii* have been used for *T. gondii* detection, including surface antigens (SAGs), dense granule proteins (dense granule antigen [GRAs]), microneme proteins (MICs), and rhoptry-associated proteins (ROPs) [16], mostly in human sera as the utilization of recombinant antigens in animals is still restricted [1]. Among these antigens, GRAs have been demonstrated to be good candidate antigens with high immunogenicity [17-20]. Consequently, they have been considered as a good serodiagnostic test for the detection of *T. gondii* antibodies both in animals and humans [21]. Many recombinant antigens have been previously studied, namely, GRA1 [21-24], GRA2 [25-27], GRA4 [11], GRA5 [19,28], GRA6 [26,29], GRA7 [18,21-24,26,30-33], GRA8 [34,35], and GRA15 [26]. GRA7 provides a very strong humoral immune response in the acute stage of infection [3] and is also recognized as a good serological marker for anti-*T. gondii* detection in the chronic stage [23,31]. Recombinant protein GRA7 has been constructed and evaluated for improving the diagnosis of *T. gondii* infection worldwide, in humans [18,36], dogs [23], cattle [30], pigs [37], chickens [21], and goats [33]; however, there has been a few reports for cats [26,31,32]. Most recombinant GRA7 proteins for *T. gondii* diagnosis are commonly performed using an *Escherichia coli* expression system. However, the expression of a heterologous protein or inappropriate conditions causes difficult/complex circumstances due to insoluble protein production or very low-level expression [38-40]. The addition of fusion tags is one of the various strategies for enhancing recombinant protein solubility and protein expression efficiency [40]. In the previous studies, fusion tags, namely, glutathione S-transferase (GST), were applied in the production of recombinant GRA7 protein as antigen for serodiagnosis of *T. gondii* infection in cats [26,32] while a small ubiquitin-related modifier (SUMO) [39,41,42] has never been used to produce recombinant GRA7 protein in the detection of *T. gondii* infection in cats.

Therefore, in this present study, we used the Expresso^®^ SUMO T7 Cloning and Expression System to produce the soluble recombinant GRA7 antigen, with the major purpose being to develop and evaluate the recombinant GRA7 protein-based indirect ELISA, hoping to utilize it as a local immunodiagnostic kit for the detection of *T. gondii* in cats in Thailand, so that this tool could be used as one of several strategies to prevent and control toxoplasmosis.

## Materials and Methods

### Ethical approval

This study involved blood collection from cats. The relevant procedures were approved by the Kasetsart University Institutional Animal Care and Committee Use (approval number: ACKU-60-VTN-007), Kasetsart University, Bangkok, Thailand.

### Study period and area

The blood collection for the study was conducted from December 2017 to June 2019. Two hundred cat blood samples were collected from the Rabies Control Division, Office of Veterinary Public Health, Health Department, Bangkok Metropolitan Administration, animal hospitals, and animal clinics (124 samples in total), and from temples (76 samples) in the Bangkok area, Thailand.

### Sample collection

Cat blood samples (1-2 mL) were collected from the saphenous vein by the veterinarian. Blood samples were kept in sterile plain tubes (no additive tube). The serum was separated using centrifugation and kept at −70°C until used.

### Recombinant construction

*T. gondii* genomic DNA was isolated from *T. gondii* tachyzoites RH strain, provided by the Department of Protozoology, Faculty of Tropical Medicine, Mahidol University, Bangkok, Thailand, using a DNA extraction kit (Geneaid, Taiwan) according to manufacturer’s instructions. Polymerase chain reaction (PCR) was conducted using the GRA7-Forward: 5’- CGCGAACAGATTGGAGGTGGTTCAGATGACGAACTGATGAGT-3’ and GRA7-Reverse: 5’- GTGGCGGCCGCTCTATTAATCTTCGCCTGATTCAGGCAC-3’ primers to amplify *GRA7* from *T. gondii* genomic DNA. Briefly, PCR components with a volume of 25 μL contained 2.5 μL of 10× ViBuffer S (160 mM (NH_4_)_2_SO_4_, 500 mM Tris-HCl, pH 9.2, 17.5 mM MgCl_2_, 0.1% Triton™X-100 and optimized with 0.35 mM of each dNTP), 2.5 μL of each primer (10 μM), 1 μL of 5 mM dNTP, 2U *Pfu* DNA polymerase (Vivantis Technologies, Malaysia), and 2 μL of DNA template. The PCR processes were pre-denaturation at 95°C for 3 min, followed by 30 cycles of denaturation at 95°C for 1 min, annealing at 60°C for 30 s, extension at 72°C for 2 min, and final extension at 72°C for 10 min. The PCR products were verified using 1.2% agarose gel electrophoresis and sent for sequencing. The *GRA7* was cloned and transformed into the pETite™ vector (pETite™ N-His SUMO Kan Vector, Lucigen, USA) and HI control 10G *E. coli*, respectively (Lucigen, USA) for recombinant construction. For protein expression, the pETite-GRA7 was extracted and cloned into HI-Control *E. coli* BL21 (DE3).

### Expression, purification, and Western blot analysis of recombinant pETite-GRA7

A single colony of *E. coli* pETite-GRA7 was inoculated in Luria Bertani Broth (LB Broth; HiMedia, India) supplement with 30 μg/mL kanamycin and incubated overnight at 37°C with stirring at 220 rpm. The culture was transferred into a fresh LB broth supplement with 30 mg/mL kanamycin and 0.5% glucose and incubated at 37°C with stirring at 220 rpm until the optical density (OD) at 600 nm reached 0.4. Then, 1 mM isopropyl β-D-1-thiogalactopyranoside was added, and the culture was further incubated at 37°C with stirring at 220 rpm for 4 h. The cell was harvested using centrifugation; then, the pellet was re-suspended with wash buffer (50 mM sodium phosphate buffer, pH 8.0 and 300 mM NaCl and 10 mM imidazole). The cell was lysed using sonication and the supernatant was collected after centrifugation. The supernatant was purified using HisTrap affinity chromatography (GE Healthcare, USA). The column was triple washed with wash buffer and the protein was stepwise eluted with elution buffer (50 mM sodium phosphate buffer, pH 8.0 and 300 mM NaCl with 100-500 mM imidazole). The protein was verified using 12% sodium dodecyl sulfate-polyacrylamide gel electrophoresis (SDS-PAGE) analysis and confirmed using Western blot with anti-6-His antibody (Sigma-Aldrich, Germany) or cat serum. For the Western blot analysis, briefly, the protein was separated using 12% SDS-PAGE and transferred onto a polyvinylidene difluoride membrane. This membrane was blocked with blocking buffer (1×Phosphate-buffered saline [PBS] buffer, pH 7.4 with 5% skim milk) at room temperature (25°C) for 2 h. Then, the membrane was triple washed with washing buffer (1×PBS buffer, pH 7.4 with 0.1% Tween 20) for 15 min. The primary antibody was diluted with washing buffer using a ratio of 1:1,000 for anti-6-His antibody or 1:500 for cat serum before being applied on the membrane. The membrane was incubated at 25°C for 1 h followed by triple washing with washing buffer for 15 min. Then 1:2000 of secondary antibody in washing buffer (goat anti-rabbit Immunoglobulin G (IgG) for anti-6-His antibody or anti-cat IgG-peroxidase antibody for cat serum) was applied and further incubated at 25°C for 1 h. The membrane was triple washed with washing buffer and the target protein was detected using SuperSignal™ West Pico PLUS Chemiluminescent Substrate (Thermo Scientific, USA).

### Indirect fluorescent antibody test

**Figure-1:**
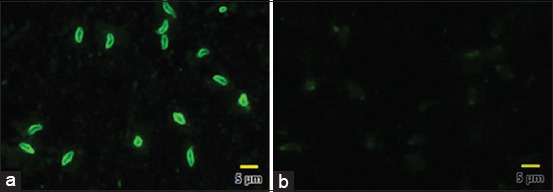
Results of indirect fluorescent antibody test in serum samples of cats. (a) Peripheral fluorescence staining is considered as positive *Toxoplasma gondii*. (b) Negative sample with no staining or only polar staining.

The antigen was produced by culturing tachyzoites of *T. gondii* RH strain as described by Udonsom *et al*. [43], provided by the Department of Protozoology, Faculty of Tropical Medicine, Mahidol University, Bangkok, Thailand. Samples of 15 mL each of the Vero cell cultures (in a concentration of 1×10^6^ tachyzoites/mL) were spotted on 6-well microscope slides (Electron Microscopy Sciences, USA) and allowed to air-dry overnight. All multi-well slides were fixed with acetone and stored at −20°C until used. The IFAT procedures were performed as described by Wiengcharoen *et al*. [44] with some modifications. Each serum sample was diluted at 1:16 [45,46] using FA Serum Diluting Buffer, pH 9.0 (VMRD, USA) for screening or two-fold from 1:32 for positive sera. Samples of 20 mL each of diluents were placed on 6-well microscope slides coated with *T. gondii* tachyzoites and the slides were incubated at 37°C for 1 h in a box containing moisture. The slides were washed for 3 min and 10 min in FA rinse buffer (VMRD, USA), respectively. Fluorescein isothiocyanate labeled goat anti-cat IgG (KPL, USA) diluted at 1:400 was added to each well on the slides before the slides were incubated again at 37°C for 1 h in a box containing moisture. After a final washing for 10 min, the microscope slides were covered with coverslips and then viewed under a fluorescence microscope at 400× magnification (Olympus, Japan). The negative and positive control sera were included when performing at the same dilutions with the samples. All slide samples and controls were carried out in duplicate. Testing was undertaken, with bright diffuse or peripheral fluorescence of the tachyzoites being considered positive, whereas a negative reaction was recorded if there was no staining or only polar staining (Figure-1), as described by Smielewska-Los *et al*. [47].

### Indirect enzyme-linked immunosorbent assay

The concentration of the pETite-GRA7 protein and the dilution of serum samples were tested to determine the optimal conditions for pETite-GRA7-based indirect ELISA. The purified pETite-GRA7 antigen was diluted with 15 mM carbonate buffer at pH 9.6 and 100 mL was immobilized on a Microplate 96-well MICROLON^®^ (Greiner Bio-One, Germany) before incubating at 37°C for 2 h. Blocking buffer (1×PBS buffer, pH 7.4 with 7% skim milk; HiMedia, India) was added to each well; then, each plate was incubated at 37°C for 1 h before adding diluted cat serum in blocking buffer. Incubation was continued for 30 min at 25°C at 300 rpm on a microplate shaker. Then, each plate was washed with washing buffer (1×PBS buffer, pH 7.4 with 0.1% Tween 20) 5 times, followed by adding diluted peroxidase-conjugated AffiniPure Goat Anti-Cat IgG (H+L) (Jackson ImmunoResearch Laboratories Inc., USA) in blocking buffer at a dilution of 1: 8000 (v/v). The plate was shaken at 300 rpm on a microplate shaker for 30 min and washed 5 times with washing buffer. After that, 3,3’,5,5’-tetramethylbenzidine (EMD Millipore Corp., USA) was added and incubated in a dark room at 25°C for 30 min. The absorbance was measured using a microplate absorbance reader at 650 nm. ELISA was performed in duplicate for all samples, with three positive and three negative controls on each plate. The cutoff was set as the mean OD of negative control sera plus three standard deviations [48]. The OD value of the blank was automatically subtracted from each sample value.

### Statistical analysis

The degree of agreement between the results of IFAT and pETite-GRA7-based indirect ELISA was measured using Cohen’s Kappa coefficient (Kappa value), with the strength of the agreement interpreted following the guidelines of Landis and Koch [49]. The sensitivity, specificity, positive predictive value (PPV), and negative predictive value (NPV) were computed for pETite-GRA7-based indirect ELISA. IFAT was the comparative test and indirect ELISA was based on pETite-GRA7 as the alternative test in all analyses. The expected test performance of ELISA at different cutoff points was evaluated using the receiver operating characteristic (ROC) curve. The accuracy of area under the curve (AUC) values was obtained as per the guideline of Swets [50] and Reynoso-Palomar *et al*. [24]. The following AUC value ranges were used with their respective accuracy interpretation: AUC<0.5, (non-informative); 0.5<AUC<0.7, (low); 0.7<AUC<0.9, (moderate); and 0.9<AUC<1, (high). Furthermore, the *χ*^2^ test was applied to test the significance between the results from the two tests. The R programming language version 4.1.0 [51] was utilized for all statistical analyses, with a 95% confidence interval (CI) and p<0.05 was considered significant.

## Results

### Genomic DNA extraction and recombinant construction

**Figure-2:**
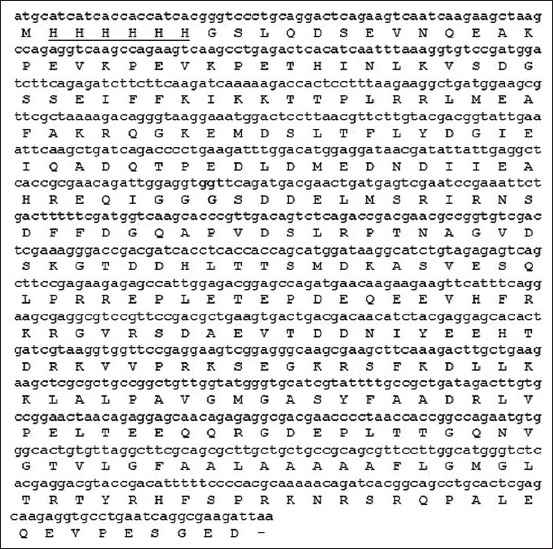
Open reading frame of pETite-dense granule antigen 7 (GRA7). Bold represents the first *GRA7* codon and underline represents 6-His tag.

A 606 bp *GRA7* PCR product was obtained using *T. gondii* genomic DNA as a template and the sequence had 100% nucleotide identity with the *T. gondii* strain RH *GRA7* gene (accession no. MK250981). An amplicon was cloned into thepETite™ vector (Lucigen) to construct pETite-GRA7 with 930 nucleotides and 309 amino acid residues (Figure-2).

### Protein expression, purification, and Western blot analysis

**Figure-3:**
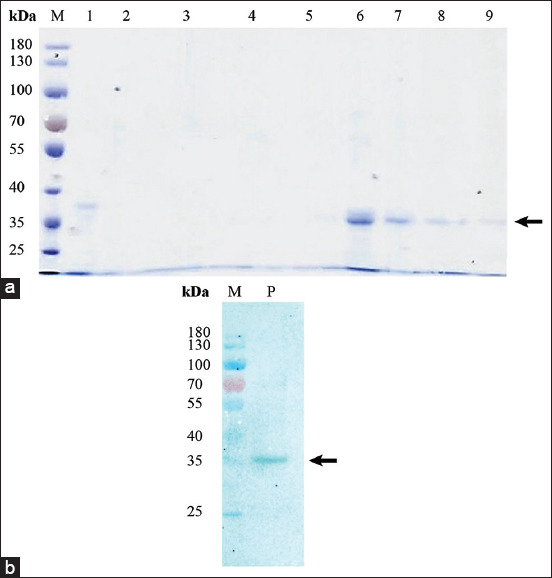
Protein expression and purification of (a) pETite-dense granule antigen 7 (GRA7) and (b) Western blot analysis against anti-6-His antibody. M=Molecular protein marker, 1=Flow through 1, 2=Flow through 2, 3=Wash 1, 4=Wash 2, 5=Elution with 100 mM imidazole, 6=Elution with 200 mM imidazole, 7=Elution with 300 mM imidazole, 8=Elution with 400 mM imidazole, 9=Elution with 500 mM imidazole, P=Purified protein. Recombinant pETite-GRA7 band indicated by black arrow.

**Figure-4:**
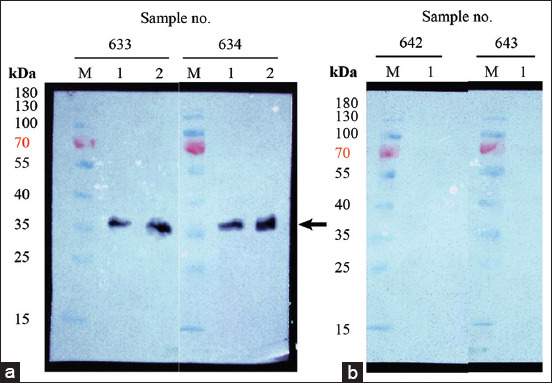
Western blot analysis of pETite-dense granule antigen 7 (GRA7) against (a) positive-*Toxoplasma gondii* cat serum and (b) negative-*T. gondii* cat serum. M=Molecular protein marker, 1=2 μg of purified protein, 2=5 μg of purified protein. Expected protein size indicated by a black arrow.

The recombinant fusion protein (pETite-GRA7) was expressed and purified. Based on the 12% SDS-PAGE, approximately 35 kDa (22.4 kDa of GRA7+12.3 kDa of pETite-fusion tag) of pETite-GRA7 was observed and confirmed by Western blot using anti-6-His antibody (Figure-3). When using anti-cat sera, the Western blot results showed positive bands against positive-*T. gondii* cat serum. In contrast, no positive band was observed when using the negative-*T. gondii* cat serum (Figure-4).

### Diagnosis of T. gondii infection in cats using ELISA with recombinant pETite-GRA7

The optimal concentration of the purified pETite-GRA7 antigen was tested at concentrations of 0.25, 0.50, 1.00, and 2.00 μg/mL together with a dilution of serum samples at 1:50, 1:100, and 1:200, as shown in Table-1. The three positive and three negative sera of *T. gondii* infection confirmed by the Department of Parasitology, Faculty of Veterinary Medicine, Kasetsart University were used for the testing. The results revealed that the OD of positive sera increased when the concentration of antigen and the dilution of serum increased, whereas changing those conditions did not affect the OD of the negative sera. Only the dilution of 1:50 was a higher OD observed using antigen concentrations of 1.00 and 2.00 μg/mL (Table-1). From the checkerboard assay, we found that a high concentration of antigen (1.00 or 2.00 μg/mL) and sample dilution (1:50) gave too high an OD; in contrast, a low concentration of antigen (0.25 mg/mL) and sample dilution (1:200) resulted in a too low OD of the sera. Thus, in our study, the pETite-GRA7 concentration of 0.50 mg/mL and the sample dilution at 1:100 was selected since they produced the most appropriate OD for the tested positive (mean OD=0.650) and negative (mean OD=0.031) sera. The dilution of secondary antibody was used at 1:8000 as it was in the range recommended by the manufacturer.

**Table-1:** Checkerboard assay to determine the optimal concentration of purified pETite-GRA7 antigen and dilution of serum samples for ELISA, expressed as the mean optical density of tested positive and negative sera.

	Antigen (µg/mL)	Sample dilution		

		1:50	1:100	1:200
Positive sera	0.25	0.464	0.260	0.141
	0.50	0.836	0.650	0.312
	1.00	1.265	0.728	0.405
	2.00	1.522	0.852	0.403
Negative sera	0.25	0.031	0.016	0.012
	0.50	0.048	0.031	0.013
	1.00	0.097	0.042	0.024
	2.00	0.132	0.053	0.023

GRA7=Dense granule antigen 7, ELISA=Enzyme-linked immunosorbent assay

In this study, a cutoff value of ELISA was set at 0.2. The testing of the 200 cat serum samples indicated that 20 samples were positive based on ELISA. Furthermore, cat sera that were identified as being infected with hookworm (n=2), *Platynosomum* spp. (n=1), *Toxocara* spp. (n=2), *Isospora* spp. (n=1), feline parvovirus (n=2), and feline immunodeficiency virus (n=1) were included in the test of indirect ELISA based on pETite-GRA7. And all of these infected samples showed negative ODs (mean OD=0.059).

### Diagnosis of *T. gondii* infection in cats using IFAT

Based on all cat serum samples, detection of *T. gondii* infection using IFAT revealed 18 positive samples at the cutoff dilution of 1:16. Under further study based on titer, all 18 positive cat sera were positive at a dilution of 1:32, with 13 samples positive with titer 1:64, while nine sera were positive at a dilution of 1:128, and only three samples were positive with titers ≥1:256 (Figure-1). In addition, all positive-*T. gondii* samples were negative with the IFAT detection of *Neospora caninum* strain NC1 (this strain provided by the Department of Protozoology, Faculty of Tropical Medicine, Mahidol University, Bangkok, Thailand).

### Comparison of results obtained using IFAT and ELISA

**Figure-5:**
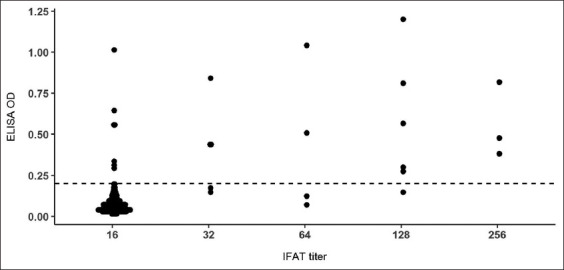
Comparison of enzyme-linked immunosorbent assay (ELISA) optical density (OD) and indirect fluorescent antibody titer in detection of antibodies against *Toxoplasma gondii* in cat sera. A cutoff point of ELISA is shown by a horizontal dashed line.

A comparison of the positive and negative results from the 200 cat sera obtained using IFAT as the comparative test and pETite-GRA7-based indirect ELISA as an alternative test indicated that 94% of samples produced the same results for IFAT and ELISA, with 87.5% concordant for negative sera. Among the total IFAT-positive sera, 27.8% were negative based on ELISA, whereas 3.8% of the IFAT-negative sera were ELISA-positive samples. Of the 18 IFAT-positive samples, 13 had a strong reaction to pETite-GRA7 with OD values in the range 0.273-1.200, while five samples produced a lower reaction with OD values in the range 0.070-0.173. In addition, of the 182 IFAT-negative samples, seven samples had a strong reaction (OD 0.293-1.014) (Figure-5 and Table-2). Seroprevalence levels of 10% (95% CI=6.6-14.9%) and 9% (95% CI=5.8-13.8%) were produced by pETite-GRA7-based indirect ELISA and IFAT, respectively. Since the prevalence percentages from the two tests were similar, the *χ*^2^ test did not indicate a significant difference between the results (*χ*^2^=0.03, p=0.86).

**Table-2:** Comparison of indirect ELISA based on pETite-GRA7 and IFAT for detection of antibodies to *T. gondii* in cat sera.

Test result	IFAT		

	No. of positives	No. of negatives	Total
pETite-GRA7-ELISA			
*No. of positives*	13	7	20
*No. of negatives*	5	175	180
Total	18	182	200

*T. gondii=Toxoplasma gondii*, GRA7=Dense granule antigen 7, ELISA=Enzyme-linked immunosorbent assay, IFAT=Indirect fluorescent antibody test

**Figure-6:**
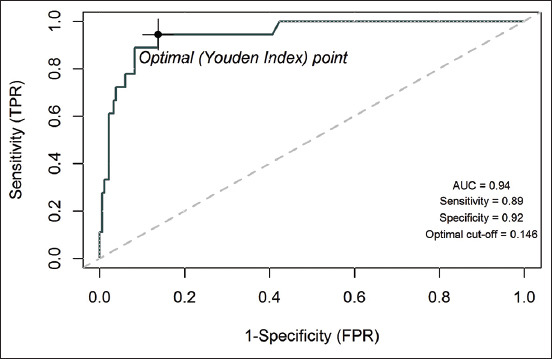
Receiver operating characteristic plot with area under the curve and optimal cutoff point providing highest sensitivity and specificity.

The Kappa value measures how much the test result from indirect ELISA based on pETite-GRA7 (at cutoff 0.2) is related to the result from IFAT (considered as true results). The Kappa value indicated that ELISA showed “substantial” agreement with IFAT (Kappa value=0.65; 95%; CI=0.46-0.84). The sensitivity and specificity of ELISA were 72% and 96%, respectively. The test results revealed a 65% PPV and a 97% NPV. The expected performance of ELISA at different cutoff points was then evaluated based on the ROC curve to determine the optimal cutoff to best distinguish between positive and negative samples while providing suitable test sensitivity and specificity. From the 200 cat serum samples, the optimal cutoff was determined using the OD of tested sera using ELISA compared to the IFAT results. The ROC-based model revealed the optimal cutoff was at 0.146 with 89% sensitivity, 92% specificity, and 0.94 of the area under the curve (AUC), which indicated a high overall assay accuracy (Figure-6).

## Discussion

Cats are only the definitive host playing an important role in the spread *T. gondii* to humans through ingestion of the sporulated oocysts in the infective stage through contaminated water or infected, uncooked meats/foods [9]. Screening based on reliable diagnostic techniques of infected cats is one of the most important ways to reduce *T. gondii* transmission from the final host to other hosts. Several serodiagnostic tests, including IHA, LAT, IFAT, MAT, DT, and ELISA [52], are applied for the detection of *T. gondii* infection in cats. ELISA is one of the most commonly used, although it is less specific [53] when the native tachyzoite antigens applied, including using these native antigens may vary significantly among laboratories or between batches; however, this limitation is removed by the development of recombinant antigen [31].

Many *T. gondii* antigens have been developed for *T. gondii* detection, such as SAG, ROP, MIC, and GRA [16,31]. SAG and GRA are seemingly used as antigenic materials in immunodiagnostic kits to detect anti-*T. gondii* antibodies while a SAG1-based ELISA test kit is commercially available for the detection of feline toxoplasmosis [54]. GRA7 is a good serological marker for anti-*T. gondii* detection has been demonstrated in cats by Cai *et al*. [31]. This superior property of GRA7 was in agreement with other researchers, such as Wang *et al*. [23], Wang *et al*. [30], Kotresha *et al*. [18], and Ybañez *et al*. [32], who have studied recombinant protein GRA7 in dogs, cattle, humans, and cats, respectively. GRA7’s ability involves the expression of this gene releasing from all infectious stages [55], mainly in tachyzoites and bradyzoites [23] with high yields presenting on the surface of host cells [23,32], in the parasitophorous vacuole (PV), PV membrane, and host cell cytoplasm [23,32,55-58]. This protein can directly connect with the host immune system, inducing strong antibody responses in the initial and late phases of infection [23,32]. With its immunodominant antigen, GRA7 could also be used for the detection of anti-*T. gondii* antibodies in the chronic and acute stages because of those antigenic properties [18,23].

The expression of *T. gondii*-recombinant GRA7 requires special conditions such as denaturation with urea or sodium dodecyl sulfate or fusion with a protein tag to obtain a high yield and high yield solubility [38]. The GST fusion protein has been commonly used to construct and express this GRA. However, such proteins require special expression conditions [26], a special buffer [59], or protease treatment [32]. In the current study, we successfully cloned and expressed GRA7 in the Expresso^®^ SUMO T7 Cloning and Expression System under standard conditions without needing any special requirements, and the use of the SUMO fusing tag also promoted the soluble pETite-GRA7 in *E. coli* and facilitated protein purification; this observation was in agreement with the study of Prejit *et al*. [39]. Some other successfully cloned genes using a similar system were recorded by Koyuncu *et al*. [60], Prejit *et al*. [39], and Hanafiah *et al*. [11].

Other researchers have evaluated the recombinant protein GRA7-based indirect ELISA in different hosts and have suggested that GRA7-indirect ELISA was a potential marker for diagnosis of *T. gondii* infection, demonstrating high sensitivity and specificity in cattle [30], patients [36], chickens [21], and dogs [23]. However, in cats there has been much less research. For example, Cai *et al*. [31] found that GRA7-ELISA was a highly accurate test with good separation between positive and negative samples. They recommended it as a promising serodiagnostic marker for the detection of infected cats with *T. gondii*. Ybañez *et al*. [32] developed an immunochromatographic test based on TgGRA7 that had high sensitivity and specificity. In the present study, pETite-GRA7-based indirect ELISA was developed for the detection of *T. gondii* in cats and compared to IFAT as the gold standard. The Kappa value showed substantial agreement between indirect ELISA and IFAT with acceptable levels of sensitivity (72%) and specificity (96%). Furthermore, no cross-reaction occurred when pETite-GRA7-ELISA was tested with other parasites or diseases. The overall performance based on ROC analysis showed that pETite-GRA7-indirect ELISA had the most appropriate sensitivity (89%) and specificity (92%) at the cutoff of 0.146 with an AUC of 0.94, which suggested a high level of accuracy for the test and its good ability to differentiate positive from negative samples. Similar findings based on ROC analysis of recombinant GRA7-ELISA were reported by Cai *et al*. [31], Sun *et al*. [21], Wang *et al*. [23], and Wang *et al*. [30]. Thus, pETite-GRA7-indirect ELISA, combined with choosing a suitable cutoff, could be useful for serological diagnosis. Furthermore, compared to IFAT, ELISA can be performed on a large number of samples at the same time, making pETite-GRA7-based indirect ELISA more useful as a good candidate for serodiagnosis of *T. gondii* infection in cats.

## Conclusion

This study showed that the SUMO expression system could be valuable in recombinant GRA7 production for anti-*T. gondii* antibody detection in cats. The developed pETite-GRA7-based indirect ELISA showed high concordant results and substantial agreement with a parallel IFAT analysis. This preliminary study demonstrated that pETite-GRA7 was a potential serodiagnostic marker leading to the development of a local pETite-GRA7-based indirect ELISA tool for diagnosis of *T. gondii* infection in cats in Thailand.

## Authors’ Contributions

BM and ES: Drafted and revised the manuscript. ES and PC: Contributed to experiments, collected and analyzed the data, and interpretation. RR and MS: Contributed to sample collection, revised the manuscript, and technical supports. SK: Contributed to some reagents/materials, technical supports, and revised the manuscript. RU: Contributed to the experiment, and technical supports. SJ: A mentor provided the guidance, supported in research, and revised the manuscript. BM: Principal researcher of the project, designed the study, contributed experiments, collected and analyzed the data, interpretation, sample collection, and finalized the manuscript for submission. All authors read and approved the final manuscript.
